# Gender differences in mathematics anxiety and the relation to mathematics performance while controlling for test anxiety

**DOI:** 10.1186/1744-9081-8-33

**Published:** 2012-07-09

**Authors:** Amy Devine, Kayleigh Fawcett, Dénes Szűcs, Ann Dowker

**Affiliations:** 1 Department of Experimental Psychology, University of Cambridge, Downing Street, Cambridge, CB2 3EB, UK; 2 Department of Experimental Psychology, South Parks Road, Oxford, OX1 3UD, UK

## Abstract

**Background:**

Mathematics anxiety (MA), a state of discomfort associated with performing mathematical tasks, is thought to affect a notable proportion of the school age population. Some research has indicated that MA negatively affects mathematics performance and that girls may report higher levels of MA than boys. On the other hand some research has indicated that boys’ mathematics performance is more negatively affected by MA than girls’ performance is. The aim of the current study was to measure girls’ and boys’ mathematics performance as well as their levels of MA while controlling for test anxiety (TA) a construct related to MA but which is typically not controlled for in MA studies.

**Methods:**

Four-hundred and thirty three British secondary school children in school years 7, 8 and 10 completed customised mental mathematics tests and MA and TA questionnaires.

**Results:**

No gender differences emerged for mathematics performance but levels of MA and TA were higher for girls than for boys. Girls and boys showed a positive correlation between MA and TA and a negative correlation between MA and mathematics performance. TA was also negatively correlated with mathematics performance, but this relationship was stronger for girls than for boys. When controlling for TA, the negative correlation between MA and performance remained for girls only. Regression analyses revealed that MA was a significant predictor of performance for girls but not for boys.

**Conclusions:**

Our study has revealed that secondary school children experience MA. Importantly, we controlled for TA which is typically not controlled for in MA studies. Girls showed higher levels of MA than boys and high levels of MA were related to poorer levels of mathematics performance. As well as potentially having a detrimental effect on ‘online’ mathematics performance, past research has shown that high levels of MA can have negative consequences for later mathematics education. Therefore MA warrants attention in the mathematics classroom, particularly because there is evidence that MA develops during the primary school years. Furthermore, our study showed no gender difference in mathematics performance, despite girls reporting higher levels of MA. These results might suggest that girls may have had the potential to perform better than boys in mathematics however their performance may have been attenuated by their higher levels of MA. Longitudinal research is needed to investigate the development of MA and its effect on mathematics performance.

## Background

Mathematics anxiety (MA) is generally defined as a state of discomfort caused by performing mathematical tasks [1]. MA can be manifested as feelings of apprehension, dislike, tension, worry, frustration, and fear [2-4]. It is not clear what factors result in the appearance of MA. Nevertheless, potential causal factors include environmental variables (e.g., negative experiences in class, teacher characteristics), intellectual variables (e.g., the degree of abstract or logical thinking) and personality variables (e.g., self-esteem, learning style, attitude and confidence [5,6]).

MA can develop in the early school years [5,7] and becomes increasingly common with age [8,9]. It is thought to affect a notable proportion of the school age population [2,10,11] and adults in post-secondary education [12]. Importantly, MA has several negative effects on children’s and adult’s mathematics education. For example, people who experience high levels of MA are likely to develop negative attitudes toward tasks involving mathematics, drop out of elective mathematics classes or avoid taking them altogether; in addition, those with high MA avoid pursuing careers that require quantitative skills [3,13-15]. This can have large-scale implications. For example, only 7% of pupils in the UK take mathematics to A level, and while there are many reasons for this, many pupils give a dislike of mathematics as a reason for not continuing [16] and sometimes the dislike is very intense and ‘charged with emotion’ [ibid, p. 10].

Some have viewed MA as form of Test Anxiety (TA) [17]. Studies have shown moderate correlations between TA and MA (between .30 and .50), so they are indeed related constructs; however, measures of MA correlate more highly with one another (between .50 to .80) than with TA, which suggests that MA is a distinct construct [2,18,19].

Of all of the negative effects that MA has on learning and using mathematics, the relationship between MA and mathematics performance has received the most attention. Past research has shown small negative correlations between mathematics performance and MA (average correlations of -.27. and -.34 in two meta-analyses) [11,19-24], indicating that those with high MA show poorer mathematics achievement. However, it has been argued that mathematics achievement, when measured in test situations, is always confounded with MA [2,25]. That is, the mathematics performance of highly mathematics anxious individuals is impaired because of their “online emotional reaction to the testing situation” [2, p. 320]. Consequently, the mathematics performance of an individual with high MA may appear lower than it actually is, when measured using a test. Furthermore, time-limited testing can negatively affect the performance of high and low maths anxious individuals, but performance is not differentially affected in the two groups [26]. However, individuals with high MA can perform similarly to individuals with low MA when mathematics problems are presented in a more relaxed format [13]. Therefore, the depressed performance associated with high MA and the reported negative correlations between MA and performance may be exaggerated because of the context in which mathematics performance is measured. Nonetheless, the effect of MA on ‘online’ mathematics performance is still pertinent, as mathematics achievement, particularly in secondary and tertiary education, is measured using time-limited tests and formal examinations. Therefore, the assessment of MA in realistic test situations is highly important as these situations exert marked influence on individual career prospects and well-being.

Further research has explored the direction of the relationship between MA and performance and two major theories have been proposed. The Deficit Theory [27], claims that anxiety emerges a result of an awareness of poor mathematics performance in the past. In contrast, the Cognitive Interference Theory [28] posits that high levels of anxiety interfere with the recall of prior learning resulting in poorer performance. A meta-analysis conducted by Hembree [19] of 151 studies of MA found more evidence to support the Cognitive Interference Theory than the Deficit Theory. However, in a more recent investigation, Birgin and colleagues found that the highest unique contribution to children’s MA was from the children’s mathematics performance [29]. Similarly, in one of the few longitudinal investigations, Ma and Xu [3] found that poorer mathematics performance led to higher MA in junior and senior high school students. Together these studies lend support to the Deficit Theory. MA resulting from an awareness of prior poor performance may be related to mathematics self-efficacy beliefs as past studies have shown that maths self-efficacy is highly predictive of MA [30-32]. The findings of two recent studies that children with diagnosed mathematical disabilities show more MA [33,34] could also indicate that poor performance leads to greater MA, though we cannot rule out the possibility that the disabilities were indeed partly caused by anxiety. Evidently the directionality of the relationship between MA and performance is open for debate and requires further research.

The relationship between gender and MA has also been studied extensively; but findings have not been consistent. There are many studies that have found significantly greater levels of MA in females than males [4,6,12,13,15,19,32,35-51]. However, there are also many studies that show no gender differences in MA [3,5,7,10,29,52-61]. There are indeed a few studies that have found higher MA levels in males than in females [62-64].

Birgin and colleagues have suggested that the lack of consistent gender effects may be because MA is not consistently defined or measured [29]. Indeed, many different MA measures have been used in past studies. The most frequently used scale is the Mathematics Anxiety Rating Scale (MARS) which has 98 items [65]. The large number of items in the scale allows the assessment of mathematics anxiety in a wide range of contexts and is therefore thought to have high construct validity. However, it requires a considerable amount of time for administration, which may make it more difficult to use with school age samples. Therefore several different shortened versions of the MARS have been developed [64,66-69], however the psychometric properties of these shortened scales have come under scrutiny [22]. Hopko and colleagues developed a 9-item scale known as the Abbreviated Math Anxiety Scale (AMAS) which was found to have strong test-retest reliability, good internal consistency and validity [22,41].

In comparison to the number of studies that have investigated gender differences in overall levels of MA, relatively few studies have explored whether the relationship between MA and maths performance or maths achievement differs by gender. Betz [12] found that correlations between MA and mathematics performance for University students differed according to gender and course: female psychology students showed a significant correlation between MA and mathematics achievement test scores, whereas males did not; in contrast, correlations between MA and mathematics achievement test scores emerged for both genders in students enrolled in an advanced mathematics course. Hembree’s meta-analysis revealed that females’ higher MA did not result in poorer mathematics performance and that MA was more predictive of maths performance in males [19]. Similarly, Miller and Bichsel [24] found that MA was more predictive of basic maths performance in males than in females; but MA was not more predictive of applied mathematics performance in either gender. Ma and Xu [3] also found gender differences in the relationship between MA and achievement. Specifically, they found that boys’ prior low maths achievement predicted later high MA at all grade levels, however girls’ prior low maths achievement only predicted later high MA at critical transition points during schooling (for example, transferring from middle school to secondary school). A possible explanation for the findings of a greater relationship between MA and achievement in males is that girls tend to experience MA whether or not they have any intrinsic difficulties in mathematics, whereas MA in boys is more likely to reflect initial problems in the subject. Alternatively, boys’ performance may be more negatively affected by anxiety, perhaps because it is less socially acceptable for them to communicate their anxieties, and thus they may be less likely to develop or be shown effective strategies of dealing with anxiety.

On the other hand, other studies have failed to find gender differences in the relationship between MA and performance/achievement [32,59]; Ma [23] gives a meta-analysis of such studies.

The general pattern of results suggests that there is a relationship between MA and maths performance or achievement, but that the direction of the relationship is not clear, partly due to the fact that studies have generally been correlational rather than longitudinal. Also, different studies have used different measures of both mathematical performance and of MA, making their results hard to compare given that some measures used may have been less reliable than others.

Given the mixed results in the field it is clear that further research, utilising reliable measures of MA, is necessary to investigate gender differences in MA and the relationship between MA and performance. The current study aims to identify whether a gender difference exists in overall levels of MA in 11- to 16-year-old children, and whether the relationship between MA and mathematics performance differs for boys and girls. The current study uses a brief MA scale, the AMAS, which is appropriate for use with young children. Furthermore the current study controls for test-anxiety which is typically not controlled for in MA studies.

It was predicted that girls would report higher MA than boys. It was also predicted that there would be a negative correlation between MA and maths performance for boys and girls, and that this correlation will be stronger for boys than girls.

## Method

### Participants

482 secondary school pupils were studied in total. 49 pupils were excluded from the investigation because they did not give at least one correct response in the mathematics test (41 pupils: 28 boys and 13 girls) or because they did not fill in the MA questionnaire (8 pupils). We decided to exclude the 41 pupils who did not fill out the mathematics test because it was hard to decide whether they did so because they were genuinely unable to solve a single task (which is very unlikely) or because they were not motivated to respond to the questions. The remaining 433 children (165 girls and 268 boys) were included in the sample: 158 children in Year 7 (mean age = 12.13 years, SD = 0.43 years), 137 in Year 8 (mean age = 13.01 years, SD = 0.44 years) and 138 in Year 10 (mean age = 15.14 years; SD = 0.40 years). Participants attended a rural comprehensive secondary school located in England, UK. The catchment area of the school was predominantly working class and lower-middle class. Participants and guardians gave appropriate informed written consent. The study was approved by the Departmental Research Ethics Committee of the Faculty of Education, University of Cambridge. The research was in compliance with the Helsinki Declaration.

## Measures and procedure

### Mathematics anxiety

The Abbreviated Math Anxiety Scale (AMAS) [22] was used to measure levels of maths anxiety. This is the shortest valid maths anxiety scale – with only 9 items, using a 5-point scale and, as mentioned earlier, has been shown to be just as effective as the longer MARS [22,41] (internal consistency: α = .90; two-week test-retest reliability: *r* = .85; convergent validity of AMAS and MARS-R *r* = .85).

### Test anxiety

Sarason’s Test Anxiety Scale [70] was used to measure test anxiety. The questionnaire contains 36 items which deal with physiological, emotional, cognitive and behavioural reactions during test-taking situations. Participants indicate whether they believe the item applies to them by answering ‘True’ or ‘False’. This questionnaire was developed many years ago. Hence, we assessed its reliability by computing Cronbach’s alpha and odd-even split-half reliability in our own sample. Cronbach’s alpha was 0.86, split-half reliability was 0.88. These values can be considered good. Hence, the TA questionnaire was reliable.

### Mathematics performance

Custom made mental mathematics tests were used in order to assess mathematical performance. Each year group was given a specific mathematics test suitable for their age range fitted to the content of their school material. The Year 7 and 8 tests each contained 20 problems and the Year 10 test contained 25 problems. The problems were written in Arabic digits rather than in words in order to minimise effects of reading problems and comprised of addition, subtraction, multiplication and division questions. We purposefully excluded rote-learned multiplication problems (i.e., common ‘times tables’) in the tests so that participants would utilize their working memory for calculation rather than simply retrieving the answers from long term memory [71]. Participants were allowed 5 minutes to complete the mental mathematics test, and were informed of this at the beginning of the session. As previous studies suggest this time pressure is not expected to have a differential effect on the performance of individuals with low and high maths anxiety [26].

All participants were tested in groups of 80 to 200, under examination conditions in their school exam hall. Each session was led by a researcher and invigilated by the participants’ teachers. Mathematics tests were administered prior to the MA and TA questionnaires.

### Statistics

Our main interest was to examine the interrelationship of MA, TA and maths performance as well as their relation to gender. Hence, in order to assure comparability across year groups, the data was standardized separately for each year group, using the mean and standard deviation of each year group. First, a Multivariate Analysis of Variance (MANOVA) was run on MA, TA and performance scores as dependent variables with factors gender (male vs. female) and school year (Year 7 vs. Year 8 vs. Year 10). Then univariate Analyses of Variance (ANOVAs) were run on each dependent variable with factors and gender and year. Other ANOVAs were run separately for girls and boys with a year factor. Pearson correlations were computed with MA, TA and performance. Partial correlations were used to control for the effect of TA. Simultaneous multiple linear regression was used to assess the relationship of mathematics performance, MA and TA. MA and TA were used as predictors of mathematics performance.

## Results

Additional file 1: Figure S1 demonstrates the MA scores of excluded participants: scores cover the whole available spectrum. In this sample the mean ± standard deviation of MA was 0.62 ± 1.35 (minimum; -1.98; maximum: 3.56) which fits the group average. The following describes the results from the main sample.

**Figure 1:**
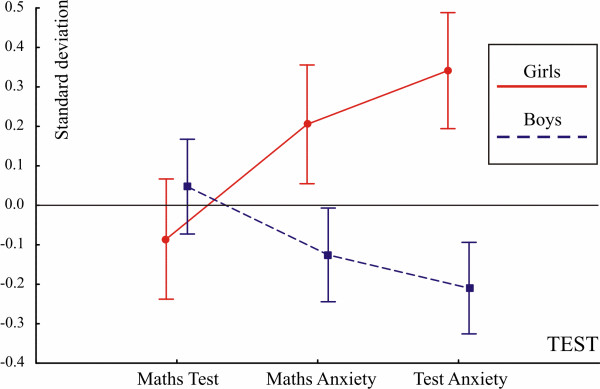
**Standardized performance scores for mathematics test performance, MA and TA by gender.** Standardized scores are in units of standard deviations (Y axis). Vertical bars denote 0.95 confidence intervals.

Gender differences on the three measures are depicted in Figure 1. The MANOVA found the gender factor highly significant (Wilks: F(3, 427) = 11.57; p <0.0001). According to univariate ANOVAs mathematics test performance was not different between girls and boys (−0.08 vs. 0.04 standard deviations; p = 0.2). In contrast, MA was 0.33 standard deviations higher in girls than in boys (0.2 vs. -0.13 standard deviations; F(2, 429) = 11.52; p = 0.0007). In addition, TA was also 0.55 standard deviations higher in girls than in boys (0.34 vs. -0.21 standard deviations; F(2, 429) = 33.51; p <0.0001). There were no other significant effects.

**Figure 2:**
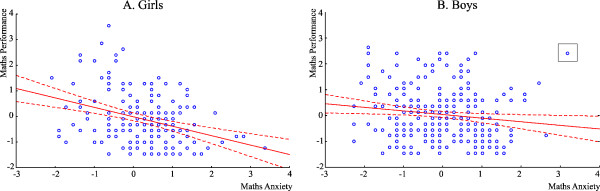
**The correlation between mathematics performance and MA for girls (2A) and boys (2B).** Mathematics performance and MA are given in standardized scores in units of standard deviations (Y axis). The box in the upper right corner marks an outlier; see text for further explanation.

**Figure 3:**
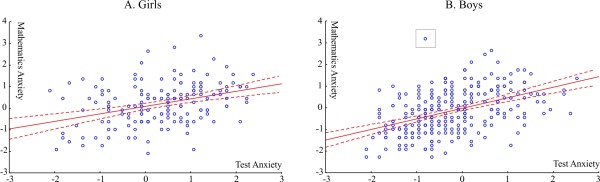
**The relationship of MA and TA in girls (3A) and boys (3B).** MA and TA are given in standardized scores in units of standard deviations. The box in [B] marks an outlier; see text for further explanation.

A single boy showed by the boxes in Figure 2B and Figure 3B showed highly outlying scores (maths performance = 2.41; MA = 3.19; TA = −0.85). Hence, this individual was excluded from correlation and regression analyses. (Nevertheless, all analyses were also run including this individual and the pattern of results did not change.) Correlation analyses established that in girls, MA was positively correlated with TA (Pearson r = 0.363; p < 0.001. Spearman R = 0.362; p <0.001) and negatively correlated with mathematics performance (Pearson r = −0.349; p <0.001. Spearman R = −0.325; p <0.001; See this relationship in Figure 2A.). TA was also negatively correlated with maths performance (Pearson r = −.207; p = 0.008. Spearman R = −0.195; p <0.05).

In boys MA was positively correlated with TA (Pearson r = .759; p <0.001; Spearman R = 0.491; p <0.001) and negatively correlated with performance (Pearson r = −0.180; p = 0.003; Spearman R = −0.172; p <0.001; See Figure 2B.). In contrast to girls, the correlation of TA and performance was only marginally significant in boys (Pearson r = −0.110; p = 0.07; Spearman R = −0.106; p <0.07). According to a difference test the strength of the correlation between MA and performance was significantly different in girls and boys (for Pearson r values: p = 0.0333).

The relationship of MA and TA in girls and boys is shown in Figure 3. The effect of TA was controlled for in partial correlation analyses. In girls MA remained strongly negatively correlated with performance (r = −.301; p <0.001). In contrast, the correlation of MA and performance was only marginally significant in boys (r = −0.108; p = 0.07). According to a difference test the strength of the MA vs. performance correlation was significantly different in girls and boys (p = 0.0233). In opposing analyses MA was controlled for. In these analyses mathematics test performance was not correlated with TA in either girls (p = 0.25) or boys (p = 0.37).

In girls the regression model (based on standardized scores) was highly significant (F(2,162) = 12.06; p <0.0001). The model accounted for 11.88% of the variance (R^2^). MA was a significant predictor variable of performance (Beta = −.315; p <0.0001). In contrast, test anxiety was not a significant predictor. In boys the overall model reached significance (F(2,264) = 3.03; p <0.05). However, it accounted only for 1.5% of the variance and neither MA, nor test anxiety emerged as significant predictors of performance.

## Discussion

We found significant negative correlations between MA and mathematics performance for boys and girls, indicating that children with higher mathematics anxiety have lower mathematics performance. These correlations support the findings of two meta-analyses which found moderate negative correlations between MA and performance (correlations of -.34 and -.27) [19,23]. In contrast, we found no difference between girls’ and boys’ mathematics performance. The lack of gender difference in mathematics performance is in line with research showing that gender differences in mathematics performance are declining, or non-existent in gender-equal countries [40,72-74].

Overall, girls reported higher levels of MA than boys, supporting our hypothesis. This finding is in line with the many other studies cited in the Introduction that found higher levels of MA in females than males, though as noted there such findings are not universal.

The reasons for why females frequently report higher MA than males is not well understood but several explanations have been offered. Some have suggested that the different ways in which boys and girls are socialized during childhood may differentially affect the anxiety experienced by males and females in certain situations [75]. This hypothesis, known as the sex-role socialization hypothesis, argues that because mathematics is traditionally viewed as a male domain, females may be socialized to think of themselves as mathematically incompetent and therefore females may avoid mathematics and when females do participate in mathematical activities they may experience more anxiety than males [75,76]. However, no link between MA and sex-role has been found and the view that mathematics is a male domain is decreasing [30,46,75,77,78]. Therefore, this hypothesis is unlikely to explain effects fully.

Another possible explanation for the gender difference in MA is that females may be more willing to admit to feelings of anxiety than males because the expression of emotion by females may be accepted whereas the expression of anxiety in males may be viewed as less acceptable [77,78]. Research has shown that females/feminine individuals are more likely to express feelings of anxiety or psychological distress than males/masculine individuals [79-81]. Flessati and Jamieson [78] tested whether gender-linked response biases can account for females showing higher MA than males by asking their participants which gender was more likely to be anxious about mathematics and other school subjects; and whether participants viewed MA as being acceptable in males, females and themselves. Contrary to the response bias hypothesis, Flessati and Jamieson found that their participants believed that MA affected both genders equally and that MA was more acceptable in males than females. Interestingly, Flessati and Jamieson found that females were more critical of the expression of MA in themselves. This finding led Flessati and Jamieson to argue that the gender difference in MA could be explained by females being more self-critical, however no follow-up research was conducted to investigate this claim.

Other variables that may account for the gender difference in MA are mathematics confidence/self-concept and mathematics self-efficacy. Several studies have shown that boys report greater confidence in mathematics than girls [76,82-86]. As mentioned previously, a similar concept, mathematics self-efficacy, has been shown to be related to MA [30,32] and boys also report higher maths self-efficacy than girls [87].

An alternative view, the maths experiences hypothesis [17] claims that gender differences in MA disappear when mathematical background is taken into account (the amount of interaction with mathematics and the number of positive/negative experiences), a finding that has been shown in college student samples [88]. However, other studies have shown that, even though maths experience is related to level of MA, maths experience does not account for the gender difference in MA [77,78].

Our study also revealed that girls showed higher TA than boys. Many of the proposed explanations for the gender difference in MA could account for the gender difference in TA too, for example, gender-linked response biases, or gender differences in self-confidence or self-efficacy. What is more interesting however is that girls showed a strong negative relationship between MA and mathematics performance, which remained even when TA was controlled for. On the contrary, boys only experienced marginal effects of general TA on performance, and when TA was controlled for, they only showed a marginal relationship between MA and performance. These results suggest that anxiety experienced by boys may simply reflect general test anxiety, whereas girls experience specific anxiety towards mathematics, which is above and beyond any general anxiety associated with testing situations. The regression model suggests MA predicts mathematics performance for girls but not for boys. Overall, these results might suggest that MA affects girls’ mathematics performance more than boys’ mathematics performance, although, we did not measure the direction of this relationship so we cannot determine whether MA affects performance or whether maths performance influenced the participants’ anxiety levels.

The finding that MA predicted performance more in girls than boys contradicts some previous findings which suggested either a greater MA-performance relationship in males, or no gender differences in this relationship. This could reflect some differences between this sample and the other (mostly American) samples that have been studied; or it could reflect the fact that other studies typically have not investigated or controlled for the effects of general TA.

However we should not forget that despite the stronger relationship between MA and performance which emerged for girls, girls’ maths performance was not significantly different to boys’ maths performance. Given that girls reported higher levels of MA than boys it is possible that girls’ mathematics performance was actually confounded by MA, or the time-limited testing procedure, and the mean score reported in the current study may not reflect the girls’ true mathematical ability: i.e., they might actually have had the potential to perform better than the boys. This is a general problem with studies of MA in relation to performance, as it is difficult to measure mathematics performance without administering a test and/or making use of existing school test scores.

Further research should involve investigating mathematics anxiety and mathematical performance longitudinally from early primary school years onwards. Some research suggests that younger children show both lower levels of MA and less of a relationship between anxiety and performance than do older children and adults [9,89]. This could be due to several possible reasons. It may be that experiences of failure or negative evaluations in mathematics lead to an increase in MA, possibly resulting in a vicious circle, which also leads to an ever-increasing MA/performance relationship. It may also be that MA increases with age for other reasons, and that it only has a negative impact on performance when it reaches a certain level of severity; indeed some have suggested an inverted U-shaped relationship between MA and performance, with moderate MA leading to better performance than no MA at all [90]. Longitudinal studies starting at an early age could give a better indication of the direction of causation, and whether it differs between the genders. It would also be desirable to use not only explicit measures of MA, but implicit measures such as arithmetic-affective priming [34] and/or measures of physiological indicators of anxiety. This could reduce the possible influence of gender differences in the willingness to report anxiety.

## Conclusions

Our study has revealed that secondary school children experience MA. Importantly, girls showed higher levels of MA than boys and high levels of MA were related to poorer levels of mathematics performance. As well as potentially having a detrimental effect on ‘online’ mathematics performance, past research has shown that high levels of MA can have negative consequences for later mathematics education. Therefore MA warrants attention in the mathematics classroom, particularly because there is evidence that MA develops during the primary school years. Furthermore, our study showed no gender difference in mathematics performance, despite girls reporting higher levels of MA. These results might suggest that girls may have had the potential to perform better than boys in mathematics however their performance may have been attenuated by their higher levels of MA. Longitudinal research is needed to investigate the development of MA and its effect on mathematics performance.

## Abbreviations

MA, Maths anxiety; TA, Test anxiety.

## Competing interests

The authors declare that they have no competing interests

## Authors’ contributions

KF and DS designed the study. KF collected the data. DS analyzed the data, contributed to the interpretation of the data and to the preparation of the manuscript. AD(1) contributed to the interpretation of the data and drafted the manuscript. AD(2) contributed to the interpretation of the data and contributed to the preparation of the manuscript. All authors read and approved the final manuscript.

## Supplementary Material

Additional file 1 **Figure S1. Standardized MA scores of excluded participants. Scores are in units of standard deviations (Y axis).** Click here for file
